# Barriers and Enablers to the Implementation of Patient-Reported Outcome Measures Within Cardiff and Vale University Health Board: A Qualitative Study of Healthcare Professionals' Perspectives

**DOI:** 10.7759/cureus.82127

**Published:** 2025-04-12

**Authors:** Gurdit Dosanjh

**Affiliations:** 1 School of Management, Swansea Univeristy, Cardiff, GBR

**Keywords:** barriers, cross-sectional, enablers, facilitators, healthcare, health policy, patient-reported outcome measures, proms, value-based healthcare

## Abstract

Background

Healthcare has experienced a shift towards outcomes-driven care. Patient-reported outcome measures (PROMs) have gained popularity as a means to achieve this goal. Cardiff and Vale University Health Board (CAVHB) is one of the seven Health Boards in Wales that has implemented PROMs in clinical care. The aim of this research was to establish the barriers and enablers that occur across the different levels (micro, meso, and macro) of the Health Board from the perspective of staff engaged in implementing PROMs. The intention is that this will provide useful insights into the important factors to consider when implementing PROMs within a healthcare setting.

Methodology

A survey on Microsoft Forms (Microsoft Corporation, Redmond, Washington) was created and sent to healthcare professionals from various departments working with PROMs in CAVHB. Multiple open-ended questions were posed to establish macro-, meso-, and micro-level enablers and barriers in the local context. Thematic analysis was carried out to generate a number of barriers and enablers from the responses.

Results

Sixteen responses were generated from a mixture of departments and disciplines. Frequently reported facilitators included the presence of a specialist Value-Based Healthcare (VBHc) and PROMs team, enthusiastic and motivated clinicians, and adequate education for patients and clinicians. The responses highlighted supportive organisational leadership and the importance of adequate resourcing for implementation efforts. IT systems and digital platforms, when poorly optimised and integrated, can act as a barrier, promote clinician fatigue, and make it difficult for patients to navigate and complete PROMs. Digital illiteracy can act as a significant barrier for segments of the population. This particularly impacted the elderly, who often lacked the ability to confidently engage with digital resources.

Conclusion

Successful implementation of PROMs must consider a number of barriers and enablers. Education is of key importance and can help mitigate several barriers that may impact clinician and patient involvement in using PROMs. IT systems must be created in a way that links with existing record systems and does not add an additional burden to clinicians. They must be easy to navigate and use for both patients and staff. Organisational buy-in is important, and adequate resourcing and staffing can help facilitate PROMs implementation.

## Introduction

There has been a shift in healthcare systems to focus on value-based, outcomes-driven care [1,2]. This drive for more value-based healthcare (VBHc) has been particularly pronounced in Wales, with the creation of concepts such as Prudent Healthcare [3] and the development of the "A Healthier Wales: Our Plan for Health and Social Care" document [4]. These initiatives highlight the political desire within Wales for a radical change in how healthcare is delivered [5,6]. In Wales, the collection and use of patient-reported outcome measures (PROMs) has been recognised as a key tool to capture relevant outcomes data since 2012 [5]. PROMs are standardised and validated questionnaires that are captured without interpretation from a clinician; they capture data from patients on their wellbeing, quality of life, symptoms and response to treatment [7-10]. PROMs data have been utilised in a number of ways, such as triaging and prioritising clinic waiting lists, improving patient-clinician communication and shared decision-making, assisting clinical decisions, evaluating healthcare practice and policy, and assisting in quality improvement [7,9,11,12].

Cardiff and Vale University Health Board (CAVHB) is one of the seven Health Boards in Wales. These Health Boards plan and deliver NHS services in their local area [13]. Since 2017, PROMs have been collected and utilised by a number of services within the Health Board, initially manually with paper forms [14]. However, since 2021, the Health Board has digitised the process with a new platform offered by "My Clinical Outcomes" (MCO), with a number of departments joining the platform [15].

Previous research in various healthcare organisations has noted a number of factors which have either hampered or facilitated PROMs implementation [8,16-18]. When introducing a change to the status quo within an organisation, it is important to explore and understand the facilitating and detracting factors for the change, as a lack of insight into these factors can decrease the likelihood of the new innovation succeeding within the organisation [19]. Therefore, for PROMs to be successfully implemented, the facilitating factors must be amplified and the barriers must be mitigated to achieve successful change.

A number of barriers are known to exist. Digital systems are acknowledged to facilitate the use of PROMs [17,20,21]; however, technology and digital platforms can create barriers, particularly when implemented poorly. The poor integration of PROMs data with existing patient electronic record systems can be troublesome [17,22,23]. Multiple digital platforms and logins can cause clinician fatigue, preventing the use of PROMs data [22]. Clinician-based barriers include poor training on how to use PROMs [17,24], clinician perceptions of PROMs being unhelpful to clinical care [22,24], clinicians fearing PROMs would be used to critique and scrutinise their practice [20], a lack of time for clinicians to interpret and act upon the data, and a lack of time to educate patients on PROMs [25,26].

Poorly educated patients are likely to interpret the questionnaires as being irrelevant to their care [22,24-26], whilst other patients may not engage due to the time-consuming nature of completing a PROM [22,24,26]. Others may struggle to engage with digital systems due to poor internet access or a lack of digital skills. Welsh Government statistics estimate that 7% of the Welsh population do not have access to the internet; this is the highest rate in the UK. Digital exclusion particularly affects lower-income adults and families, the disabled, those with chronic health issues, and the elderly [27]. This can act as a barrier to using PROMs, especially as these groups are more likely to have contact with the healthcare system [6]. Poor direction and guidance from senior leadership can also hamper implementation efforts [7,24].

Adjusting care pathways to include PROMs as an important, routine aspect of healthcare provision is a facilitator for the use of PROMs [7,17]. Engaging clinical staff in creating these pathways and empowering them to implement PROMs can motivate people to engage with the collection and utilisation of PROMs [16,24]. Importantly, integrating PROMs data into existing electronic record systems helps to reduce clinician fatigue [6,16,17,22]. Adequate resourcing, such as sufficient numbers of staff assisting with the collection of PROMs and adequate IT facilities, has been found to enable PROMs implementation [8,16,28-30]. Ensuring that both clinicians (such as by adding PROMs-related topics in nursing and medical education) and patients receive education on PROMs can support increased engagement [8,17,22,24,29]. Another important patient-related factor is selecting the right PROM so that the target patient population finds relevance in its questions, and ensuring they have easily accessible help if they encounter difficulties with completing their PROM [24].

Little evaluation of the overall success of implementation has been carried out in the Welsh context. It is unknown whether there are any unique local factors that impact the implementation of PROMs. Also, with the increased drive for digital collection of PROMs within Wales and CAVHB, it is useful to understand whether digital systems produce any additional barriers that affect PROMs implementation. These data would help guide the creation of more robust digital PROMs collection systems, in Wales but also across the globe. Hence, identifying and understanding the enablers and barriers to PROMs implementation within the CAVHB will benefit organisations within Wales. It will also support clinical systems across the world in the preparation and implementation of PROMs within their own paradigms, by offering an example for comparison with their own implementation processes.

Aims and objectives

The aim of this cross-sectional study was to understand the micro-, meso-, and macro-level barriers and enablers for PROMs implementation within the CAVHB by gathering insights and perspectives from clinical staff (such as physicians, nurses, physiotherapists, and healthcare assistants) within the Health Board.

## Materials and methods

As mentioned, the study was conducted in CAVHB, with staff working within the Health Board. A two-week period between July 25, 2024, and August 8, 2024, was set for data collection. The data were collected via a survey. The survey was created on Microsoft Forms and was developed by the author of this paper. It was designed to gather specific qualitative information on micro-, meso- and macro-level enablers and barriers within the CAVHB system. Descriptions of these three levels can be found in Table 1. Research shows that healthcare systems operate on the aforementioned three levels. The sum of the barriers and enablers across these levels determines organisational success in achieving change [31]. Therefore, the questions of the survey were designed to ensure these three areas were addressed in the responses.

**Table 1:** Descriptions of the structural levels a healthcare system operates at

Level	Description
Micro	The clinical team level
Meso	The organisational level
Macro	The health system level

Healthcare professionals were contacted via the CAVHB VBHc team, who had links with departments involved with PROMs. The VBHc team provided the contacts for staff from these departments, who were then contacted via email. The VBHc team did not select specific participants. The participants were invited to complete an anonymous and voluntary questionnaire conducted via Microsoft Forms. Two emails were sent out: an initial contact and a subsequent follow-up reminder email one week later.

Some brief demographic questions were included to understand personal characteristics of the respondents, such as their role, department, and experience with PROMs (to confirm experience implementing and using PROMs within CAVHB).

This was followed by further questions utilising Likert scales for quantitative responses on how participants felt about the overall implementation process in the CAVHB, along with open-text qualitative questions to explore and expand on the Likert scale responses, and to allow respondents an opportunity to freely document their thoughts and experiences. Questions on particular barriers and enablers within their teams and departments were posed, and free text was utilised to give participants the opportunity to detail their experiences without restriction. Further themes were explored through open questions to understand the meso- and macro-level barriers and enablers, particularly in relation to organisational and legislative issues surrounding PROMs implementation.

The questionnaire underwent an assessment for accuracy through pre-testing during the development phase of the survey, with colleagues external to the CAVHB who are also involved in VBHc initiatives. This was done to ensure the validity of the survey by confirming that the questions were clear and sufficient to gather useful information aligned with the study objectives. Minor changes to the wording of the questions were made following piloting to improve clarity.

A thematic analysis of the survey data was utilised to identify patterns and allow for detailed analysis of the qualitative aspects of the data. This is a widely accepted method for analysing qualitative data [32]. It provided a methodical and structured approach to analysis. The data analysis was carried out by the author. The initial step in the process was coding the responses. This was done using inductive coding; no pre-defined codes were utilised. The raw data were evaluated, and recurring points in the survey responses were noted. This helped to assign a label, or code, to similar points in the dataset. The codes were then reviewed and grouped into overarching, higher-level themes. This process helped to identify common trends and patterns in the data. The themes were then analysed to determine whether they represented barriers or enablers. Microsoft Excel (Microsoft Corporation, Redmond, Washington) was utilised to organise the various codes and themes during the analysis.

Ethics approval was obtained via the Swansea University ethics committee. Informed consent to participate in the study was implied by participation. A statement on consent was included with the survey. It highlighted that completion of the survey implied the participant’s consent to be included in the study, and that consent could be withdrawn at any time by contacting the researcher.

## Results

Study participants 

A total of 16 people responded out of 32 contacted. This represented a 59% response rate. All respondents had been actively involved in implementing PROMs, with the majority currently using or actively rolling out PROMs in their department. Table 2 summarises the demographic information of participants, including their department and job role.

**Table 2:** The employment characteristics of the participants

Department	N (%)
Myeloma service	2 (12.5)
Neuroendocrine tumours service	2 (12.5)
Cardiology department	5 (31.25)
Mental health service	4 (25)
Neurology department	2 (12.5)
Lymphoedema service	1 (6.25)
Job role	N (%)
Nurse	8 (50)
Occupational therapist	2 (12.5)
Psychologist	1 (6.25)
Physician	5 (31.2)

Fifteen of the survey participants were currently involved with using PROMs, with only one respondent having previously been involved with implementing and using PROMs.

Responses to questions

There was a Likert scale with three statements regarding the overall experience of implementing PROMs in CAVHB, with options ranging from overall negative experience (N = 4), a mixed experience (N = 12), and an overall positive experience (N = 0). Most indicated an experience with a mix of positive and negative elements, suggesting that both frustrating and enabling factors were at play in CAVHB.

The next question was an open-text question, asking: "Were there any particular barriers that challenged your team in implementing and using PROMs?"

Half (N = 8) of the respondents noted poor integration between patient electronic record systems and the MCO platform being used for PROMs collection, with one respondent lamenting that it was "an extra program to log in to on top of other portals". A quarter (N = 4) of the respondents also mentioned that digital illiteracy and lack of internet access among some patient groups affected a department's ability to utilise PROMs. Further IT-related issues were reported, with three responses citing problems with the MCO platform itself, particularly a poor user interface. One respondent noted they had issues with secure internet access, which hindered PROMs completion. A summary of the responses to this question can be seen in Table 3.

**Table 3:** The key barriers noted following thematic analysis of the survey data The table shows the barrier and description of the barrier alongside quotes from participants.

Theme – Barriers	Comments	Quotations
Resistance from staff	Two participants (N = 2) reported that they felt their team members experienced unacceptable levels of disruption by having to utilise PROMs, with some suggestion of poor integration with existing clinical routines being one of the causes driving this	“Some resistance from more experienced staff”; “Some nurses did not use it often as it was very separate to their workflow”
Interoperability issues between the digital PROMs collection platform and clinical systems	Half (N = 8) of the users experienced some level of poor synchronisation and integration between existing electronic systems, such as the Welsh Clinical Portal (WCP) and PARIS. PARIS is CAV’s mental health clinical record system and database. This caused a poor user experience, with some users reporting reduced accessibility to PROMs data as a result of poor optimisation with existing electronic record systems	“Initial difficulty with our electronic notes system. Onerous task for IT staff to embed on PARIS system”; “An extra program to log in to on top of portals”; “User experience was not integrated into the workflow requiring staff to log into another system to view”; “Lack of integration into clinical systems”; “Results not integrated in WCP, unable to see all answers”; “Needs to be more accessible for clinicians and integrated to WCP”; “No time in clinic to download the patient results on top of other clinical outcomes”
Lack of support staff	Two (N = 2) respondents mentioned the poor availability of support staff, who could assist patients in completing PROMs, as being a barrier to their completion	“Not all measures used are easily accessible by patients (e.g. because of learning/cognitive capacity) and therefore without support to make sense of the questions they are not answered honestly”; “No admin support or HCSW staff to help patients complete them (PROMs) if they are unable to do so independently”
Digital illiteracy amongst target patients	A quarter (N = 4) of respondents reported that a poor understanding of digital systems acts as a barrier to patients interacting with PROMs, which are predominantly recorded on an electronic system. This barrier was particularly reported amongst certain departments which featured a higher proportion of elderly patients, suggesting certain patient demographics are more likely to be excluded by the digital shift	“High cohort of elderly patients… (some are) unable independently”; “Elderly population who don't have access to QR codes or simply don’t understand or able to go online to complete PROMS”; “Very elderly ones struggle a bit due to technical barriers”
Poor patient experience with the PROMs collection platform (MCO)	Three (N = 3) participants reported patients experiencing problems utilising the MCO platform, with some facing particular issues signing up to the platform and being unable to complete the relevant PROM due to this	“Some patients struggled to register”; “The set up of new patients could have been better”; “The current platform is not very user friendly from our point of view”
Physical infrastructural issues	Only one (N = 1) respondent reported regular issues with their allocated outpatient and clinic facilities, with internet and IT issues causing disruption and inability to complete PROMs	“IT/Internet issues in outpatients/clinic prevented patients from completing PROMS on the day”;
Lack of support from senior leadership/wider organisation	Two (N = 2) respondents felt there was poor direction from leadership on how to implement PROMs	“Lack of top-down support in many areas to support use of PROMS”; “It was difficult to get the appropriate guidance”
The data are not intelligently presented to clinicians on the PROMs collection software, causing poor insights	Clinicians (N = 2) felt there was trouble with MCO as a platform, particularly on how data was presented and how it could be interpreted	“To be able to accumulate data and analyse it would be much more useful”; “Viewing PROM scores was not intelligent (on the platform)”
Clinicians feel data from PROMs is not always useful for clinical care	Two (N = 2) respondents felt that PROMs themselves were not a useful tool for impacting patient care. This is less of a CAV-specific barrier and more of an issue with PROMs themselves	“Doesn’t always alter care provided”; “Looks at a specific point in time… doesn’t always capture a client’s full therapeutic experience”
Difficult to send digital PROMs links due to no requirement/difficulty accessing patient information, such as emails	One respondent (N = 1) mentioned poor access to patient data, such as emails	“Poor access to patient emails”

The next question was: "Were there any enablers or supportive aspects during your adoption process which enabled your implementation and use of PROMs?" A number of themes emerged in this section as well. Many respondents (N = 5) noted the importance of experienced, motivated and enthusiastic staff who understood the purpose of PROMs and were keen to utilise them. Having a sufficient number of staff, such as health support workers, to complete and assist patients with completing PROMs, and for the wider multi-disciplinary team to appreciate the importance of PROMs in clinical practice, was noted to be of benefit (N = 5). Another facilitator mentioned was the availability of funding and support to acquire "iPads" and computers for use in clinic areas (N = 2). Supportive leadership was recognised to underpin these enablers, as respondents said that their leadership acknowledged the need for adequate funding and resourcing (N = 2). Another popular facilitator was the presence of the PROMs team/VBHc team based in CAVHB, who had offered support and guidance for PROMs implementation and supported implementation efforts (N = 6). Linked to this was the fact that departments had access to nationally licensed PROMs and could request access to the PROMs needed (N = 1). A summary of the responses for this section can be seen in Table 4.

**Table 4:** The key enablers noted following thematic analysis of the survey data The table shows the barrier and description of the barrier alongside quotes from participants.

Themes – Enablers	Comments	Quotes
Available and supportive CAVHB VBHc team	The presence of the VBHc team was a commonly reported enabler, with six respondents (N = 6) offering positive sentiments regarding the team	“Good support and guidance from the VBHc team”; “We were well supported”; “Good CAV PROMs team”; “During implementation the team were available and very helpful”
Good choice and accessible PROMs	The ease of access to certain PROMs (N = 1) was noted as a positive aspect of PROMs implementation in CAV	“We were able to request the PROMs we needed”
Enthusiastic, knowledgeable and motivated staff and colleagues	Five respondents (N = 5) mentioned that their team members recognised PROMs as important to adopt and use, and having experienced and knowledgeable staff was noted as beneficial for implementation efforts	“My team were very enthusiastic about value based care and getting patients involved. Had particularly driven colleague who spoke to us about it and educated us on the potential application of the PROMs in other health boards”; “Majority of staff recognised it was important to adopt”; “Supportive team”; “Previous experience and dept expertise with disease specific PROMS”
Supportive senior leadership	Two respondents (N = 2) mentioned having the support of senior staff as being useful for guidance and obtaining funding to be able to implement PROMs	“Senior staff also recognised and so obtaining funds and training was relatively easy”; “We had regular meetings arranged both at Health Board level and with the National team”
Availability of support staff	Five respondents (N = 5) noted that a key enabler was the availability of support staff to assist with patients with PROMs completion, and the wider multi-disciplinary team recognising and appreciating the importance of PROMs in clinical practice	“When we have had administrative support to enter in the answers for the PROMs”; “Outpatient staff when able would show patients how to complete form and access them”; “Enthusiastic outpatient department staff helping patients complete PROMS”; “Support from the outpatients team, who recognised the importance of PROMs and understood why we did it”
Good IT support available	Two respondents (N = 2) mentioned they had good IT support	“IT support in setting up service”; “Able to get us iPads and tech for use in clinical areas when completing PROMs”

The next question sought to understand whether any specific support was provided by the Health Board itself to adopt PROMs. Four respondents (N = 4) reported that no specific organisational-level support was provided, while the remaining (N = 12) indicated that some level of support had been given. The subsequent question asked for clarification on the nature of support offered, if the respondent had answered positively. The key importance of support from the PROMs team was highlighted by half of the respondents (N = 8). A further few respondents (N = 2) mentioned regular check-ins and meetings from local management and leadership to assess progress. Two respondents (N = 2) also mentioned that teaching sessions and materials had been provided to their department for PROMs implementation.

The survey sought to understand whether there were any specific micro-level issues at the patient level impacting PROMs implementation, by asking how patients had responded to PROMs and whether any barriers or facilitators had influenced patient attitudes. Ten respondents (N = 10) indicated themes that acted as barriers to patient engagement. These included patients perceiving PROMs questionnaires as repetitive and refusing to engage with repeated collections (N = 3). Others reported that patients experienced a lack of meaningful outcome from completing PROMs, did not understand the purpose of PROMs (N = 3), and did not appreciate their role in clinical care (N = 2). Digital illiteracy impacted some patients' ability to engage (N = 4), with the MCO platform being described as difficult to navigate (N = 3). Six responses (N = 6) mentioned patient-level enablers for PROMs implementation. A younger patient demographic (N = 1) was considered more likely to complete PROMs reliably. Well-educated and informed patients who understood the role of PROMs in their healthcare journey were more likely to complete them consistently (N = 4). Another enabler (N = 1) was the habituation of patients to complete a PROM as a routine part of the care provided by the service.

A final question sought to understand whether macro-level factors, such as government policy or legislation, had impacted respondents' ability to implement PROMs. The majority (N = 14) indicated that no specific factors at this level had played a role. The responses received focused on PROMs licensing, with two (N = 2) alluding to national PROMs licensing as an enabler for the use of PROMs in clinical practice.

## Discussion

This cross-sectional study evaluated the implementation of PROMs in CAVHB by engaging a number of colleagues across departments and from various roles within the Health Board.

Overall, the respondents indicated having both positive and negative factors impacting their implementation journey within CAVHB. It is useful to turn to the distinction of micro-, meso-, and macro-level factors to separate out the various elements found in the research. Distinguishing between the barriers and enablers by using this system-level form of categorisation may make it easier for those wishing to adopt PROMs to understand that issues must be addressed at each level to achieve meaningful change. Further research in other Health Boards in Wales, or more detailed comparisons between departments within CAVHB itself, can be used to explore micro- and meso-level factors in more detail and understand how they impact the effectiveness of PROMs implementation, as has been done in studies assessing other non-PROMs-related areas of policy implementation [33].

The vast majority of issues faced by CAVHB staff were at the micro and meso levels. Education of clinicians, support staff and patients can support implementation efforts, whilst a lack of it can hamper such efforts. A team to support implementation, such as a PROMs team like the one in CAVHB, can prove to be of great use to those seeking guidance. While there is a push for an increasingly digitised healthcare landscape, which can have great advantages, it is important to develop digital tools in ways that support workflows and do not add additional barriers for clinical staff. Patients who lack the resources or skills to engage with technology must not be excluded from efforts to modernise systems. A study by Nilsen et al. found that changes in healthcare organisations were more likely to succeed if staff had opportunities to influence change, if they were adequately prepared for the change, and if they valued the change [34].

In the CAVHB research, at the micro level, both adequate education and an appreciation of the importance of PROMs in clinical practice were noted to be powerful enablers for successful change. Staff who were surrounded by enthusiastic and motivated colleagues who could educate and motivate others created a conducive environment for the implementation of PROMs. Previous experience of implementing and using PROMs provided experiential knowledge that could be shared with colleagues. Conversely, staff who struggled to identify the role of PROMs in clinical care and did not understand their purpose were noted to be barriers. It is imperative that staff tasked with implementing changes in practice are supported in gaining the knowledge and skills required to utilise the innovation and to understand its role in improving service provision. This is part of ensuring adequate preparations are made for introducing change to the status quo.

Although the study did not directly assess patient views, clinical staff suggested some patient-level factors. Patients completing PROMs must have a good understanding of how PROMs can improve their care. Respondents mentioned that some patients found PROMs repetitive, suggesting this as a reason they struggled to complete them. Collecting multiple PROMs over time can help monitor the progression of a condition or the effect of treatment [35]. It may be that patients were not educated on this benefit of PROMs and therefore found the process cumbersome and repetitive. This highlights the importance of education and of involving patients in their own care. An estimated 7% of the Welsh population do not have access to the internet [27]. The research from this study noted that many patients, particularly elderly patients, have struggled to complete PROMs. Certain departments, with higher proportions of vulnerable patients, may be at increased risk of their patients being affected by digital exclusion. A heavy digital focus can impact the right to equitable access to healthcare for all citizens and must be considered when implementing new technologies in clinical settings.

At the meso level, the organisation should support implementation efforts. It is known that factors such as adequate resources being allocated to support the change, having the right infrastructure, and buy-in from managers and leaders within an organisation are useful meso-level factors that can enable implementation [19,36]. The research reinforces these findings. Leadership and management must see the benefit of providing resources to clinical departments to mitigate the effects of barriers such as Wi-Fi or computer issues, to ensure adequate staffing to assist patients in collecting PROMs data, and to create electronic platforms that are easy to operate and provide data in easy-to-digest formats.

CAVHB utilises a number of different patient administration systems, such as the nationally used Welsh Clinical Portal (WCP) and PARIS, CAV’s mental health clinical record system and database [37]. As has been noted, these systems are separate from the MCO platform. This prevents data from being collated on one system and prevents the formation of accurate dashboards. This limitation reduces how beneficial PROMs can be to key stakeholders, such as clinicians and service managers. IT issues featured prominently in the responses. There was frustration regarding poor integration of the various computer systems and difficulty interpreting the data on the platform. MCO also appeared to be difficult for patients to operate, with difficulty accessing the correct PROMs. These platforms must be designed with users in mind. A poorly designed and poorly optimised system can turn what should be an enabler for PROMs use into a barrier, by being a frustrating and cumbersome tool. This demonstrates how IT is a bi-directional factor and can be either an enabler or a barrier if not implemented thoughtfully.

The presence of a specialist PROMs team was frequently mentioned as helpful for staff implementing PROMs. Such a coordinating role within a healthcare organisation has been noted as having a beneficial impact on clinical teams implementing PROMs in other studies, with assistance provided to resolve IT issues, support in interpreting and analysing PROMs data, and engagement across the different levels of the healthcare system by communicating with stakeholders throughout the organisation [8]. This study confirms the central role of having such a team to provide guidance and facilitate implementation.

The research did not find many factors at the macro level. This refers to the level of national policy, organisations and legislation. Due to the nature of this study, it was not possible to explore in detail why this was the case. Sparse findings at this level could indicate limited insight or understanding of macro-level influences among frontline staff, who may find meso- and micro-level factors more impactful and visible in their day-to-day work. A minority of responses referred to national PROMs licensing as a facilitating factor. One national macro-level enabler is the establishment of organisations such as the Welsh Value in Health Centre, the Centre for Healthcare Evaluation, Device Assessment and Research (CEDAR), and the NHS Wales Informatics Service. These organisations aim to develop digital and technological solutions to healthcare problems and to support the development of digital platforms for PROMs collection [6]. As part of this role, organisations such as CEDAR have contributed to the licensing of PROMs and the identification of appropriate tools for use across the country. Licensing PROMs tools on a national level helps to standardise the measures in use and allows for consistency across the system, assisting in benchmarking across clinical groups [38]. Certain PROMs have licensing fees. This cost aspect must also be considered [1,38]. Negotiating this at a national level may provide cost benefits and may be another reason why national PROMs licensing is beneficial.

Strengths and limitations

Most previous research on PROMs implementation has focused on one department or disease. This study captured a spectrum of departments and disease states, allowing for the collection of experiences from stakeholders across various disciplines. It is also the first noted study of its kind within a Welsh context. This will be useful for drawing comparisons with other organisations and healthcare settings.

In terms of limitations, it is noted that the study features a small sample size of sixteen respondents. This may impact the generalisability of the research, as it may not be representative of the larger population, and it can increase the risk of bias in the responses, such as skewing the data towards a certain speciality if that group is over-represented. This could be seen in that the research mainly captured respondents in medical specialties rather than surgical ones. Another possible limitation is that it is difficult to determine whether the data reached saturation due to the limited sample size. In spite of this, certain themes did recur a significant number of times, suggesting that the study was able to draw some meaningful conclusions. Factors such as poor interoperability of the IT systems were mentioned by half of the respondents, while other factors, such as the availability of the CAVHB VBHc team or having motivated and enthusiastic colleagues supporting implementation, appeared frequently within the data. Another limitation is that the respondents self-selected for participation in the study. This could bias the data, as respondents who were particularly engaged and enthusiastic about PROMs, or those who were particularly frustrated by the implementation process, may be over-represented, having been more likely to engage with the survey. Nevertheless, these perspectives remain insightful and can assist in highlighting important factors, as this study does, in improving the implementation of PROMs in the Welsh healthcare setting.

Despite the limitations, relevant insights were gained through this study on PROMs in a novel setting, with many findings consistent with previous research studies in other contexts. This helps confirm that, in spite of the small sample size, meaningful findings were identified. The study may be instructive for further studies on the implementation landscape in Wales and the wider healthcare community.

## Conclusions

In this cross-sectional study, commonly reported facilitators for PROMs implementation included the presence of a specialist VBHc and PROMs team, enthusiastic and motivated clinicians, and adequate education. IT can make PROMs collection more efficient; however, platforms must be carefully designed to avoid causing clinician fatigue and should be integrated with existing record systems. Digital illiteracy can also act as a significant barrier for a section of the population. Education is crucial to encourage involvement from both clinicians and patients in using PROMs as part of their care. These findings are largely consistent with prior research and highlight the importance of considering these factors when implementing PROMs in a new healthcare setting.

## Appendices

**Table 5:** Survey questionnaire

Question	Response
What service/department do you work in?	
What is your role within the department?	
What is your experience with PROMs?	Currently rolling out PROMs/Attempted to use in the past but have stopped/Currently successfully using PROMs in clinical practice/Worked with PROMs in a previous department/Other, please write
On the scale below, please mark what your overall experience has been with the implementation process with PROMs:	Positive/Mixed/Negative
Please explain your choice above	
Were there any particular barriers that challenged your team in implementing and using PROMs?	
Were there any enablers or supportive aspects during your adoption process which enabled your implementation and use of PROMs?	
Did the Health Board provide any encouragement/support to your department for adopting VBHc practices such as utilising PROMs?	
In your opinion, how have patients reacted to PROMs? Are there any patient-level barriers or enablers impacting the uptake of PROMs?	
Has any Government/Trust rules/legislation affected your use of PROMs?	Yes/No
If yes, please explain	
